# On the Simulation of Ultra-Sparse-View and Ultra-Low-Dose Computed Tomography with Maximum a Posteriori Reconstruction Using a Progressive Flow-Based Deep Generative Model

**DOI:** 10.3390/tomography8050179

**Published:** 2022-08-24

**Authors:** Hisaichi Shibata, Shouhei Hanaoka, Yukihiro Nomura, Takahiro Nakao, Tomomi Takenaga, Naoto Hayashi, Osamu Abe

**Affiliations:** 1 The Department of Radiology, The University of Tokyo Hospital, 7-3-1 Hongo, Bunkyo-ku, Tokyo 113-8655, Japan; 2 The Department of Computational Diagnostic Radiology and Preventive Medicine, The University of Tokyo Hospital, 7-3-1 Hongo, Bunkyo-ku, Tokyo 113-8655, Japan; 3 The Center for Frontier Medical Engineering, Chiba University, 1-33 Yayoi-cho, Inage-ku, Chiba 263-8522, Japan

**Keywords:** computed tomography, deep learning, image reconstruction, maximum a posteriori, unsupervised learning, X-rays

## Abstract

Ultra-sparse-view computed tomography (CT) algorithms can reduce radiation exposure for patients, but these algorithms lack an explicit cycle consistency loss minimization and an explicit log-likelihood maximization in testing. Here, we propose X2CT-FLOW for the maximum a posteriori (MAP) reconstruction of a three-dimensional (3D) chest CT image from a single or a few two-dimensional (2D) projection images using a progressive flow-based deep generative model, especially for ultra-low-dose protocols. The MAP reconstruction can simultaneously optimize the cycle consistency loss and the log-likelihood. We applied X2CT-FLOW for the reconstruction of 3D chest CT images from biplanar projection images without noise contamination (assuming a standard-dose protocol) and with strong noise contamination (assuming an ultra-low-dose protocol). We simulated an ultra-low-dose protocol. With the standard-dose protocol, our images reconstructed from 2D projected images and 3D ground-truth CT images showed good agreement in terms of structural similarity (SSIM, 0.7675 on average), peak signal-to-noise ratio (PSNR, 25.89 dB on average), mean absolute error (MAE, 0.02364 on average), and normalized root mean square error (NRMSE, 0.05731 on average). Moreover, with the ultra-low-dose protocol, our images reconstructed from 2D projected images and the 3D ground-truth CT images also showed good agreement in terms of SSIM (0.7008 on average), PSNR (23.58 dB on average), MAE (0.02991 on average), and NRMSE (0.07349 on average).

## 1. Introduction

X-ray chest computed tomography (CT) is a three-dimensional (3D) image modality. It has diagnostic superiority over chest X-rays (CXRs), but patients have greater radiation exposure than in the case of CXRs [1]. To reduce radiation exposure, sparse-view CTs have been developed. Typical sparse-view CTs adopt a maximum a posteriori (MAP) reconstruction, which can reduce the number of projection images for CT reconstruction. Those sparse-view CTs adopt a prior that assumes a sparsity of images, e.g., regularization terms of quadratic form in [2] and the $l_{1}$ norm in compressed sensing [3]. Sparse-view CTs are used to reconstruct a 3D image from tens of two-dimensional (2D) projection images, but Shen and coworkers [4,5] proposed ultra-sparse-view CT algorithms to reconstruct a high-resolution 3D image from a single or a few projection images. A similar work by Ying et al. [6] reconstructed a high-resolution 3D CT image from biplanar CXR images. The typical resolution of previous methods for a reconstructed 3D image is 128×128×128. However, previous algorithms related to ultra-sparse-view CT [4,5,6,7,8] adopt end-to-end supervised deep neural networks without exception: those algorithms do not handle MAP reconstruction, in which log-likelihood and cycle consistency loss are simultaneously optimized. Instead, those algorithms minimize a loss function which contains mean absolute errors between the ground truth images and reconstructed 3D images. Note that pure deep learning methods for supervised learning cannot handle MAP reconstruction because they cannot compute log-likelihood. The lack of optimization of log-likelihood means that there is no explicit guarantee that those algorithms can reconstruct images that are likely to be the 3D ground-truth CT images. The lack of the optimization of the cycle consistency loss means that there is no explicit guarantee that the reconstructed 3D image projected onto a 2D plane coincides with the input 2D projection image. These missing factors can potentially deprive these ultra-sparse-view CT algorithms of robustness against noise. The lack of robustness is especially problematic in ultra-low-dose protocols, where strong noise significantly contaminates the 2D projection images.

**Figure 1:**
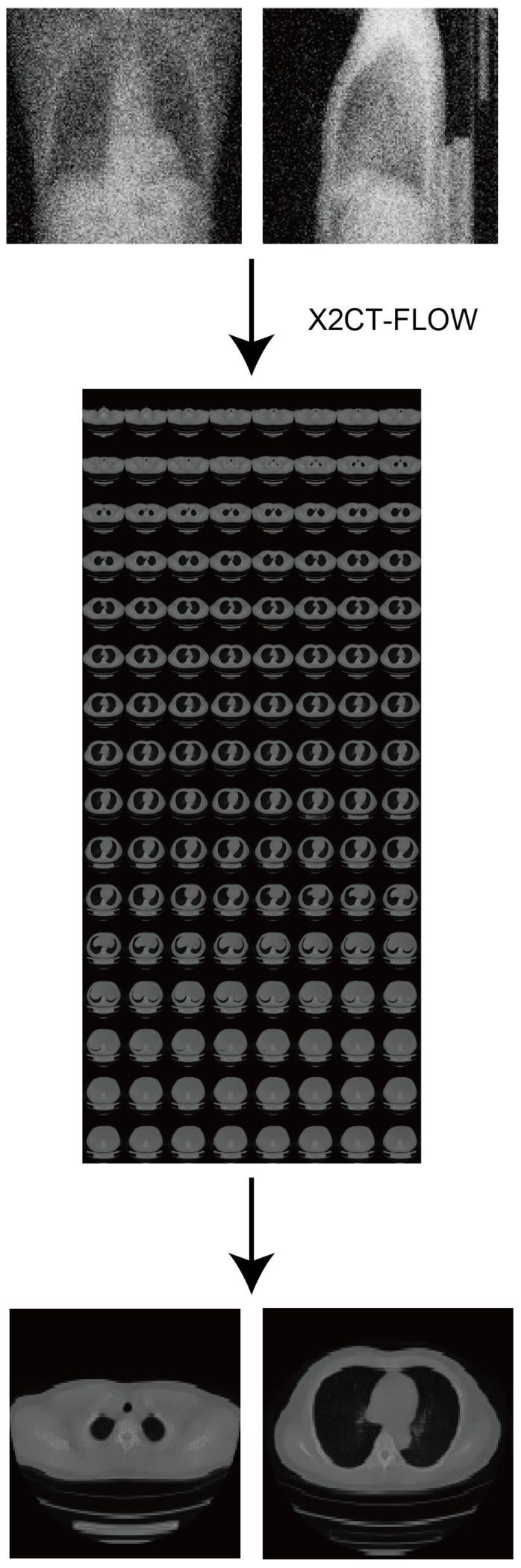
X2CT-FLOW can find the optimum 3D chest CT image (the **middle** and **bottom**) from a single or a few noisy projection images (the **top**) with MAP reconstruction. Scales of the images are not the same.

Here, we propose a novel ultra-sparse-view algorithm especially for simulated ultra-low-dose protocols (**X2CT-FLOW**, Figure 1), which adopts the MAP reconstruction. Unlike ordinal compressed sensing, we do not explicitly impose sparsity on reconstructed images for a prior with the regularization terms; instead, we train the prior with a progressive flow-based deep generative model with 3D chest CT images. The MAP reconstruction can simultaneously optimize the log-likelihood and the cycle consistency loss of a reconstructed image in testing (for details, see Section 2). We built the proposed algorithm on 3D GLOW developed in our previous study [9], which is one of the flow-based deep generative models; the models can execute exact log-likelihood estimation and efficient sampling [10]. Furthermore, we realize training with high-resolution ($128^{3}$) 3D chest CT images with progressively increasing image gradations (**progressive learning**), and showcase a high-resolution 3D model. To the best of our knowledge, there is no previous study of the flow-based generative models in which such a high-resolution model was showcased.

In summary, the contributions of this paper are as follows:

1. We propose the MAP reconstruction for ultra-sparse-view CTs, especially for simulated ultra-low-dose protocols, and validate it using digitally reconstructed radiographs.
2. We establish progressive learning to realize high-resolution 3D flow-based deep generative models.
3. We showcase a 3D flow-based deep generative model of 3D chest CT images, which has state-of-the-art resolution ($128^{3}$).

## 2. Materials and Methods

### 2.1. Materials

This retrospective study was approved by the ethical review board of our institution, and written informed consent to use the images was obtained from all the subjects. We used chest CT images of 450 normal subjects. This dataset contains only 1 scan per subject. These images were scanned at our institution with a GE LightSpeed CT scanner (GE Healthcare, Waukesha, WI, USA). The acquisition parameters were as follows: number of detector rows, 16; tube voltage, 120 kVp; tube current, 50–290 mA (automatic exposure control); noise index, 20.41; rotation time, 0.5 s; moving table speed, 70 mm/s; body filter, standard; reconstruction slice thickness and interval, 1.25 mm; field of view, 400 mm; matrix size, 512 × 512 pixels; pixel spacing, 0.781 mm. We empirically noticed that 3D GLOW fails to learn images if the number of images in the training dataset is not enough. Therefore, in contrast to usual machine learning approaches, we randomly divided the images of the 450 normal subjects into training (384), validation (32), and test datasets (34).

### 2.2. Pre-Processing

To make it easier to train our model, we reduced the image gradation from 16 bits to 8 bits. Specifically, we converted the acquired images $I_{\mathrm{src}}$ (CT number in HU units) into images $I_{\mathrm{dst}}$ with the following empirical formula:

(1) $$\begin{array}{c} I_{\mathrm{dst}}=\frac{255\cdot \left\{\mathrm{clip}\left[I_{\mathrm{src}},-1000,\max\left(I_{\mathrm{src}}\right)\right]+1000\right\}}{\max\left(I_{\mathrm{src}}\right)+1000}, \end{array}$$

where the operator clip(x,a,b) restricts the value range of an array *x* from *a* to *b*, and the operator max(x) returns the maximum value in *x*.

We introduced a 2D projection image vector $y_{i}^{j}$ whose dimensions are $H_{2D}\times W_{2D}\times C_{2D}$ and a 3D chest CT image vector $x_{i}$ whose dimensions are $D_{3D}\times H_{3D}\times W_{3D}\times C_{3D}$, where $H_{2D}$, $W_{2D}$, and $C_{2D}$ are the height, width, and channel size of the 2D image and $D_{3D}$, $H_{3D}$, $W_{3D}$, and $C_{3D}$ are the depth, height, width, and channel size of the 3D image, respectively. The subscript *i* distinguishes patients and we omit it if not necessary, and the superscript *j* distinguishes different view angle images for each patient, where 1≤j≤N and *N* is the number of the angles, e.g., N=1 for a uniplanar (single) image and N=2 for biplanar images. To simplify the explanation below, we set N=1; hence, we omit the superscript *j*. We show formulations in cases of N≥2 in Appendix C.

We first trained a flow-based deep generative model (3D GLOW) using a set of 3D chest CT images, and then reconstructed a 3D chest CT image from a single or a few 2D projection images with a latent space exploration (X2CT-FLOW). Owing to limits in GPU memory, we downsampled $I_{\mathrm{dst}}$ to the resolution of $128^{3}$; hence, we set $D_{3D}=H_{3D}=W_{3D}=H_{2D}=W_{2D}=128$ and $C_{3D}=C_{2D}=1$.

### 2.3. 3D GLOW

**Figure 2:**
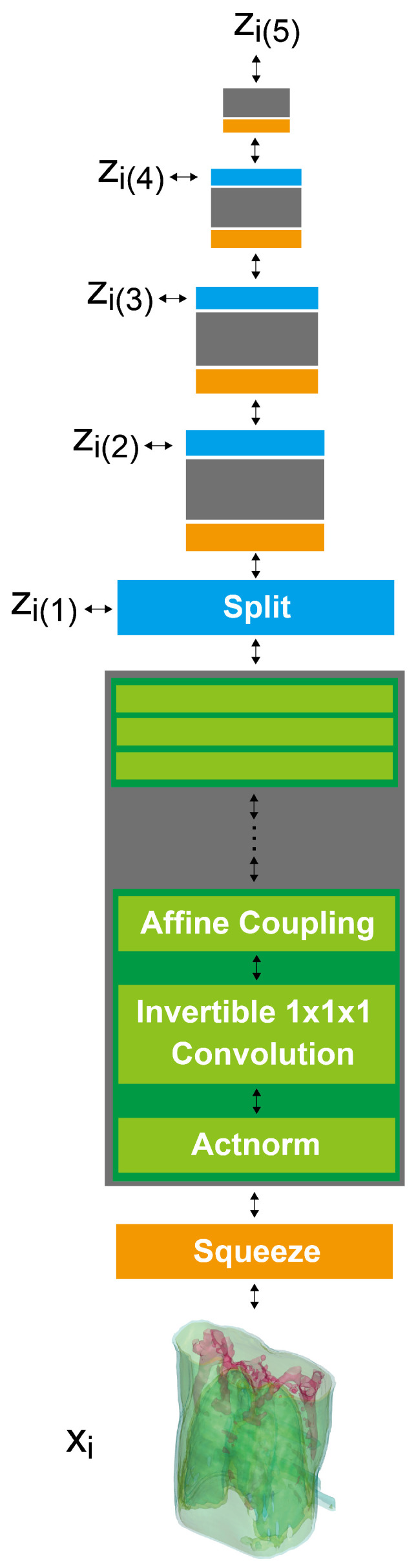
Deep neural network architecture of 3D GLOW. $x_{i}$ represents a 3D CT image vector and $z_{i(k)},k=1,\ldots ,5$ represent the latent variable vectors in each deep neural network level. We rendered the 3D chest CT image with three iso-surfaces. X2CT-FLOW explores the latent variable vectors $z_{i(k)}$ to generate the optimum 3D CT image vector ($x_{i}$).

In training, the flow-based deep generative models minimize the Kullback–Leibler divergence between the true distribution $\left[p(x_{i})\right]$ and the estimated distribution $\left[p_{\theta }\left(x_{i}\right)\right]$ of input images (i.e., 3D chest CT images) by minimizing the negative log-likelihood (NLL) as

(2) $$\begin{array}{ccc} L\left(D\right) & = & -\frac{1}{\left|D\right|}\sum_{x_{i}\in D}\log p_{\theta }\left(x_{i}\right), \end{array}$$

where the subscript θ represents the parameters in the model, D represents a set of images for training, |D| is the number of images for the training, and the subscript *i* distinguishes each image. The NLL is not tractable; therefore, we map the NLL onto a tractable simpler distribution (e.g., a multivariate independent normal distribution) as:

(3) $$\begin{array}{c} \log p_{\theta }\left(x_{i}\right)=\log p\left(z_{i}\right)-\log\left|\det\left(\frac{\partial G_{\theta }}{\partial z_{i}}\right)\right|, \end{array}$$

where $p\left(z_{i}\right)$ is the tractable probability density function, e.g., the standard normal distribution $z_{i}∼N\left(0,I\right)$, and $x_{i}=G_{\theta }\left(z_{i}\right)$ is the invertible decoder in the model. We adopted 3D GLOW developed in our previous study [9], which is a 3D extension of one of the state-of-the-art 2D flow-based deep generative models, GLOW [11]. We indicated the concrete form of $G_{\theta }$, i.e., the deep neural network architecture of 3D GLOW, in Figure 2. GLOW enabled the fake but realistic image generation by introducing invertible 1 × 1 convolution, which is a kind of flow permutation, in addition to an affine coupling layer.

Here, for the first time, we propose to train the flow-based deep generative models in a progressive manner to accelerate the convergence of the NLL. Firstly, we trained 3D GLOW with 2 bits images and then 3 bits, 4 bits, and finally 8 bits images in whole the training dataset. We explain the details of our progressive learning in Appendix A. Moreover, we show the beneficial effects of the progressive learning in Appendix B.

By using a trained 3D GLOW model, we can generate fictional but realistic images, i.e., sampling, as follows:

(4) $$\begin{array}{ccc} z_{i} & ∼ & N\left(\mu_{\theta },T^{2}\cdot \Sigma_{\theta }^{2}\right), \end{array}$$

(5) $$\begin{array}{ccc} x_{i} & = & G_{\theta }\left(z_{i}\right), \end{array}$$

where *T* (scalar) is the temperature for the reduced-temperature model, i.e., we can sample from the distribution $p_{\theta ,T}\left(x\right)\propto {\left[p_{\theta }\left(x\right)\right]}^{T^{2}}$ [12], $\mu_{\theta }$ is the estimated means of the images for training in the latent space, and $\Sigma_{\theta }^{2}$ (diagonal matrix) is the estimated variances of the images for training in the latent space. For details of the flow-based deep generative models, see [11,13,14].

To further enhance the stability of the training of 3D GLOW, we modified the scale function in the affine coupling layer to the scale $s\left(h_{2}+2.0\right)+\epsilon$ from the scale $s\left(h_{2}+2.0\right)$, where *s* is the sigmoid function, $h_{2}$ is the input from the previous split layer, and ϵ is a newly introduced hyperparameter. We empirically set $\epsilon =10^{-3}$. We introduced this hyperparameter to further stabilize the training by preventing the division by zero.

The hyperparameters used to train the model are listed in Table 1. We utilized Tensorflow 1.14.0 for the back end of the DNNs. The CUDA and cuDNN versions used were 10.0.130 and 7.4, respectively. All processes were carried out on a workstation consisting of two Intel Xeon Gold 6230 processors, 384 GB memory, and five GPUs (NVIDIA Quadro RTX 8000 with 48 GB memory). For the training, we only used four GPUs out of the five GPUs, and for the testing, we utilized only one GPU.

### 2.4. X2CT-FLOW

In testing, we reconstructed the 3D image from a single or a few noisy 2D projection images by exploring the latent variable vectors z to generate the optimum 3D CT image vector x. We define a linear observation matrix *P* as follows:

(6) $$\begin{array}{ccc} y_{h,w,c} & = & {\left(Px\right)}_{h,w,c} \end{array}$$

(7) $$\begin{array}{ccc} & \equiv & \frac{1}{D_{3D}}\sum_{d=1}^{D_{3D}}x_{d,h,w,c}, \end{array}$$

where the indices d,h,w, and *c* distinguish voxels and the observation matrix *P* is a linear operator to average voxels in the depth direction. We can similarly define the observation matrices for different projection directions. First, we adopt the matrix to emulate 2D projection images y obtained with an ultra-sparse-view CT from an image x obtained with a standard CT, i.e., forward projection. In this study, we did not use 2D projection images obtained with an ultra-sparse-view CT because these do not exist. Second, we adopted the matrix to reconstruct x from y, i.e., back projection. We found $\hat{x}$ such that it maximizes the log-posterior of x given the observation fact y, i.e., logp(x|y). We created y so that the probabilistic distribution of noise on y follows a normal distribution $\left[N(0,\sigma^{2}I)\right]$. Therefore, we have

(8) $$\begin{array}{ccc} y & = & Px+\sqrt{\sigma^{2}}w, \end{array}$$

(9) $$\begin{array}{ccc} w & ∼ & N(0,I), \end{array}$$

where $\sigma^{2}$ is the variance of the normal noise (scalar) and w is a normal noise vector. Equation (8) means that logp(y|x) follows a normal distribution for fixed x and *P*. Using the above definitions, we finally have

$$\begin{array}{ccc} \hat{x} & = & \underset{x}{\arg\;\max}\log p\left(x|y\right)\\ & = & \underset{x}{\arg\;\max}\log p\left(y|x\right)+\log p\left(x\right)-\log p\left(y\right)\\ & = & \underset{x}{\arg\;\max}\log p\left(y|x\right)+\log p\left(x\right)\\ & = & \underset{x}{\arg\;\max}\log\left[\frac{1}{\sqrt{2\pi \sigma^{2}}}\exp\left(-\frac{1}{2}w^{T}w\right)\right]+\log p\left(x\right)\\ & = & \underset{x}{\arg\;\max}-\frac{1}{2}\log2\pi \sigma^{2}-\frac{1}{2\sigma^{2}}{∥y-Px∥}_{2}^{2}+\log p\left(x\right) \end{array}$$

(10) $$\begin{array}{ccc} & = & \underset{x}{\arg\;\max}-\frac{1}{2\sigma^{2}}{∥y-Px∥}_{2}^{2}+\log p\left(x\right) \end{array}$$

(11) $$\begin{array}{ccc} & \equiv & \underset{x}{\arg\;\max}-E\left(x\right), \end{array}$$

where between the first and the second lines, we applied Bayes’ theorem.

The first term of Equation (10) is the cycle consistency loss and the second term of Equation (10) is the log-likelihood term. We approximate the log-likelihood term $\left[\log p(x)\right]$ by $\left[\log p_{\theta }\left(x\right)\right]$ using a trained 3D GLOW model. Moreover, we empirically replaced the log-likelihood term $\log p_{\theta }\left(x\right)$ with $\log p_{\theta }{\left(x\right)}^{T_{b}^{2}}$, where $T_{b}^{2}={\left(\log2\cdot D_{3D}\cdot H_{3D}\cdot W_{3D}\right)}^{-1}$, i.e., bits per dimension.

On the basis of Equation (10), we iteratively reconstructed the optimum 3D chest CT image from each chest 2D projection image in a testing dataset. We adopted the gradient descent method to obtain $\hat{x_{i}}$ such that it can satisfy Equation (10), i.e.,

(12) $$\begin{array}{c} x_{i}^{\left(n+1\right)}\leftarrow x_{i}^{\left(n\right)}-\alpha \cdot \nabla_{x_{i}}E\left(x_{i}^{\left(n\right)}\right), \end{array}$$

where α is an empirical relaxation coefficient and the superscript *n* is an iteration number. Furthermore, to accelerate the convergence of Equation (12), we adopted an invertible decoder $G_{\theta }$ of 3D GLOW, which can map a latent vector $z_{i}$ to a 3D chest CT image $x_{i}$, i.e., $x_{i}=G_{\theta }\left(z_{i}\right)$. Finally, we adopted the gradient descent method to obtain $\hat{z_{i}}$ such that $\hat{z_{i}}$ can satisfy Equation (10), i.e.,

(13) $$\begin{array}{c} z_{i}^{\left(n+1\right)}\leftarrow z_{i}^{\left(n\right)}-\alpha \cdot \nabla_{z_{i}}E\left[G_{\theta }\left(z_{i}^{\left(n\right)}\right)\right], \end{array}$$

and if the $l_{2}$ norm between the current latent vector $z_{i}^{\left(n+1\right)}$ and the previous latent vector $z_{i}^{\left(n\right)}$ converges, we can obtain the optimum 3D chest CT image ${\hat{x}}_{i}$ as

(14) $$\begin{array}{c} {\hat{x}}_{i}=G_{\theta }\left({\hat{z}}_{i}\right). \end{array}$$

### 2.5. Validations

During the training of 3D GLOW, we monitored the averaged NLL for the validation dataset. We stopped the training and saved the model when the NLL took its local minima. Then, we qualitatively and statistically validated the reconstruction performance with X2CT-FLOW by adopting a set of unseen projection images in the test dataset. For the statistical evaluation of the reconstruction performance, in addition to the mean absolute error (MAE; the lower is the better) and normalized root mean squared error (NRMSE; the lower is the better), we prepared the means and variances of structural similarity (SSIM; the higher is the better) [15] and peak-signal-to-noise-ratio (PSNR; higher is better) between reconstructed 3D images and the ground-truth images, as in [6]. SSIM can quantify similarity between two images. PSNR can quantify degradation between two images.

## 3. Results

### 3.1. Standard-Dose Protocol

We assume the limit of $\sigma^{2}\to 0$. In this limit, we have

(15) $$\begin{array}{c} E\left[G_{\theta }\left(z\right)\right]\to \frac{1}{2\sigma^{2}}{∥y-PG_{\theta }\left(z\right)∥}_{2}^{2}. \end{array}$$

We put $\alpha =0.2\sigma^{2}\cdot \left[1-\exp\left(-0.01\cdot n\right)\right]$ and iterated while n≤1000 and $∥y-PG_{\theta }\left(z\right){∥}_{2}^{2}>3^{2}\cdot N\cdot H_{2D}\cdot W_{2D}$.

**Figure 3:**
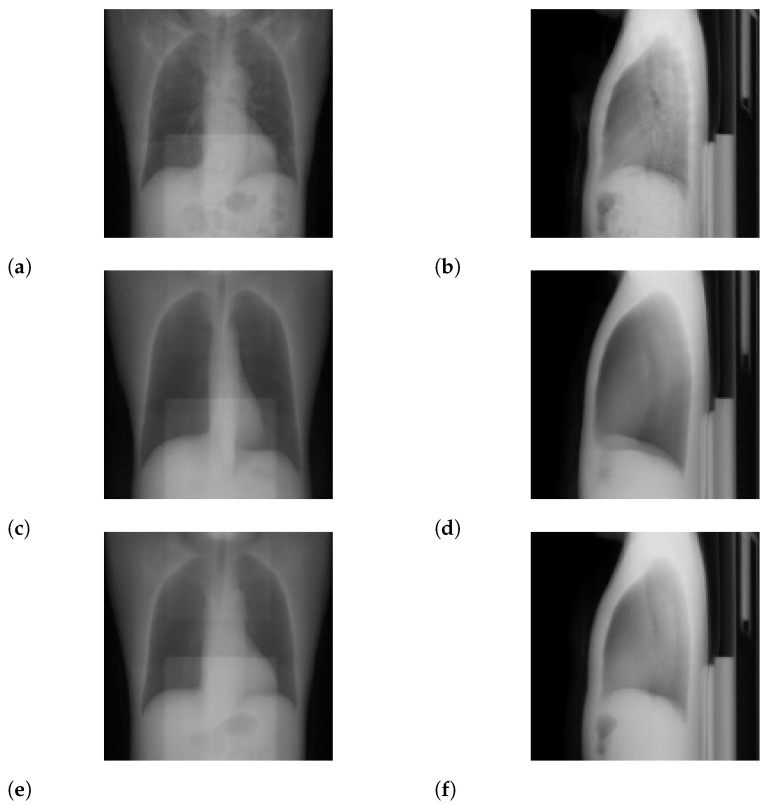
Input images and projections of a reconstructed image (N=2): (**a**,**b**) input images, (**c**,**d**) projections of an initial guess image (sampled with temperature T=0.5), (**e**,**f**) projections of the optimum reconstructed image. The intensities of these images were modified to enhance visibility.

**Figure 4:**
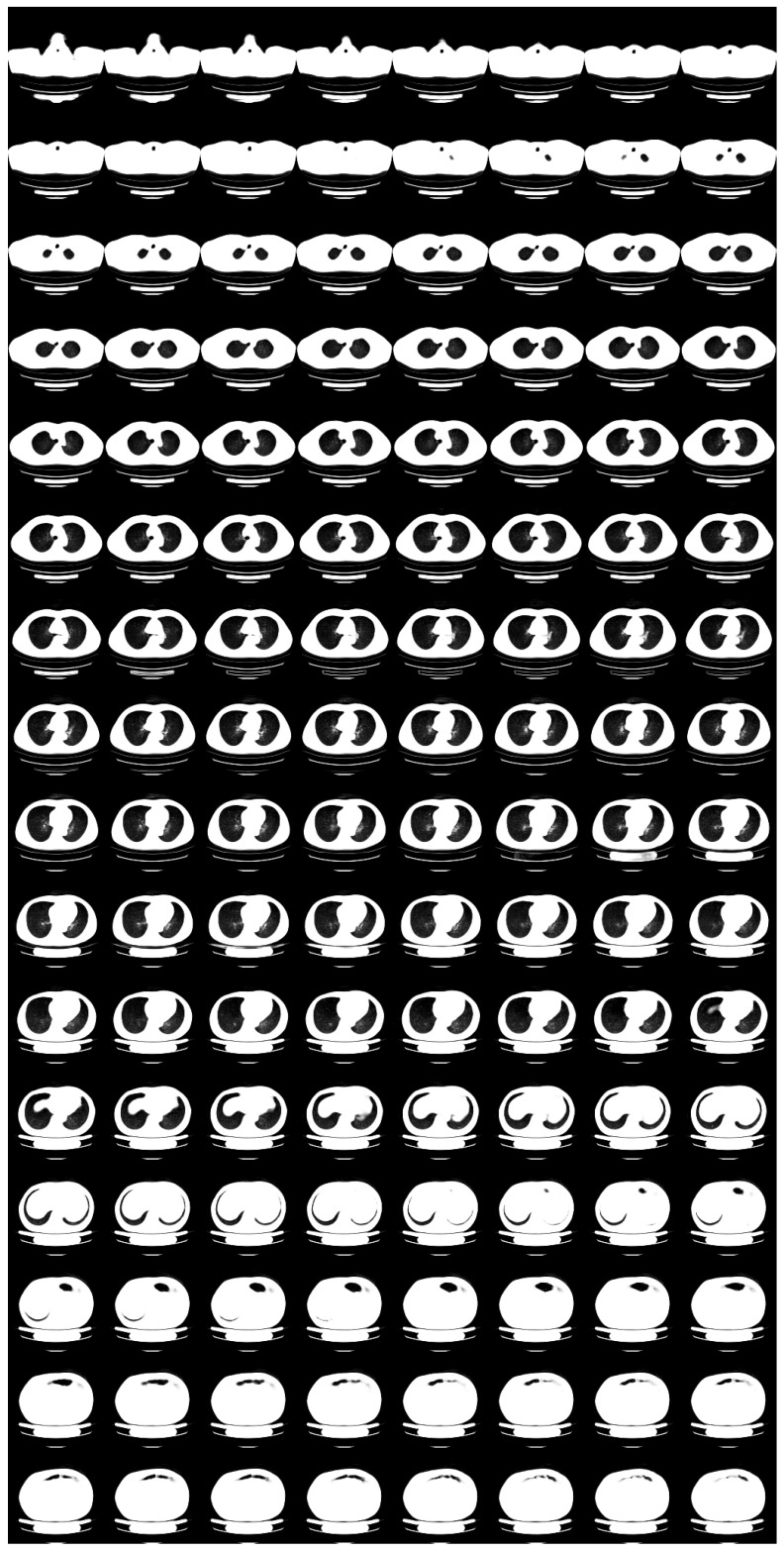
Reconstructed 3D CT image with X2CT-FLOW from Figure 3a,b ($\sigma^{2}=0,N=2$), in pulmonary window setting.

**Figure 5:**
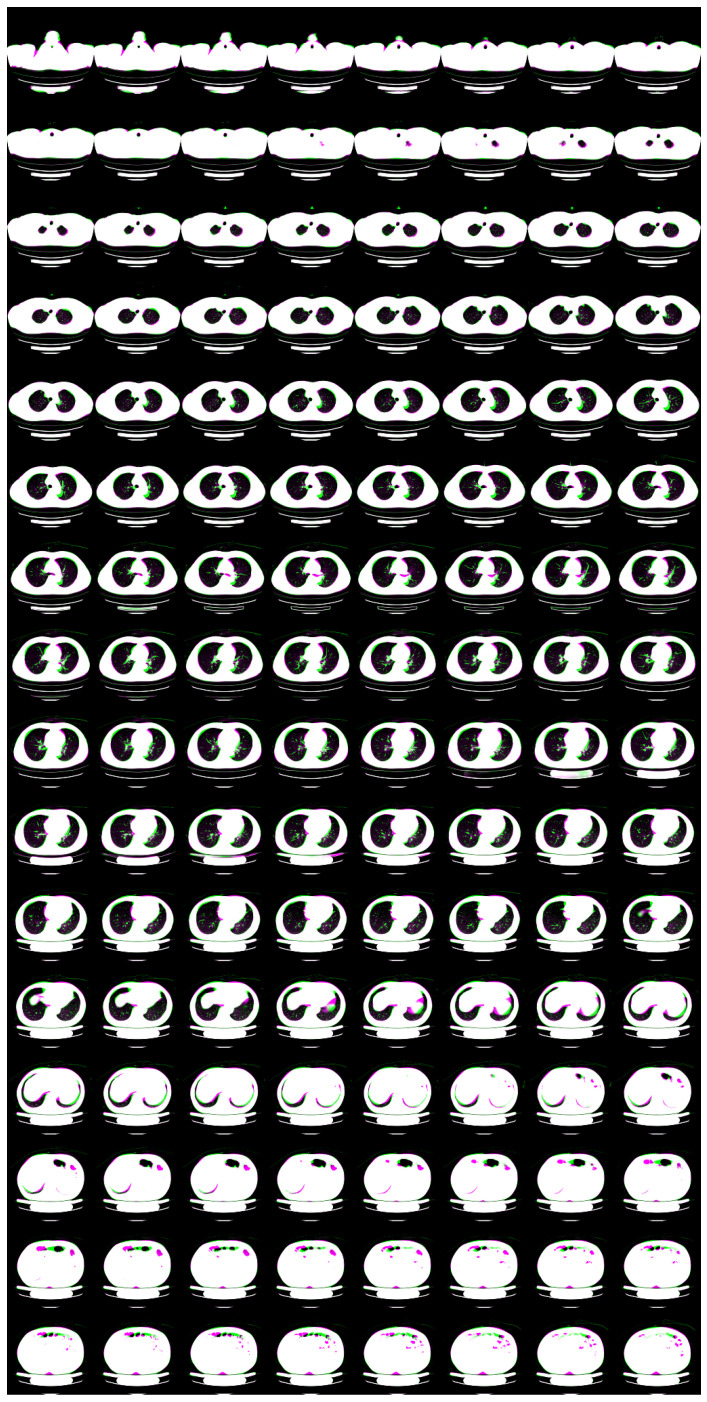
Superposition of the reconstructed 3D CT image shown in Figure 4 (magenta) and the ground-truth image (green).

**Figure 6:**
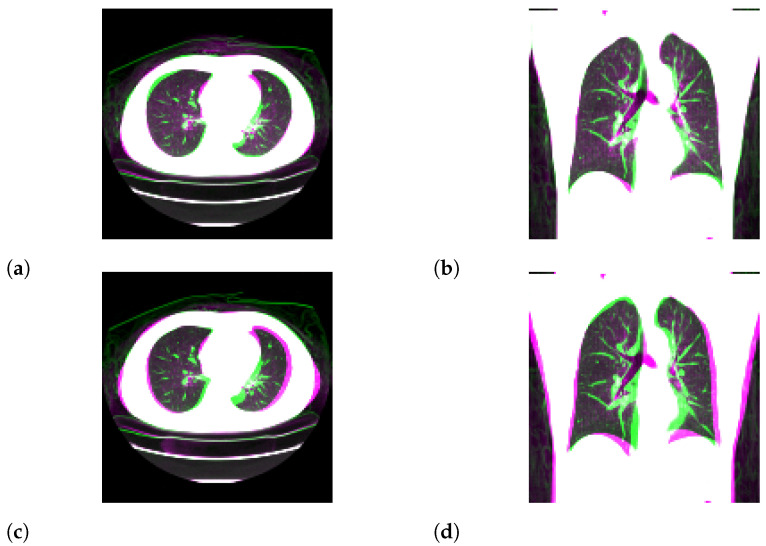
Superposition of the reconstructed 3D CT image (magenta) and the ground-truth image (green) in pulmonary window setting. (**a**,**b**) Partially enlarged axial and coronal views of Figure 5. (**c**,**d**) Partially enlarged axial and coronal views of Figure 9.

For N=2, we show input 2D images without noise and 2D projections of 3D reconstructed images in Figure 3. Moreover, we show a 3D chest CT image reconstructed from Figure 3a,b in Figure 4 and a differential image between the reconstructed 3D image and the ground-truth image in Figure 5. We show enlarged axial and coronal slices in a pulmonary window setting in Figure 6.

For N=1 and N=2, we show the means and variances of SSIM, PSNR, MAE, and NRMSE between the reconstructed 3D chest CT images and ground-truth images in Table 2 and Table 3. Moreover, we show our results with X2CT-GAN [6] trained with our materials explained in Section 2.1.

### 3.2. Ultra-Low-Dose Protocol

**Figure 7:**
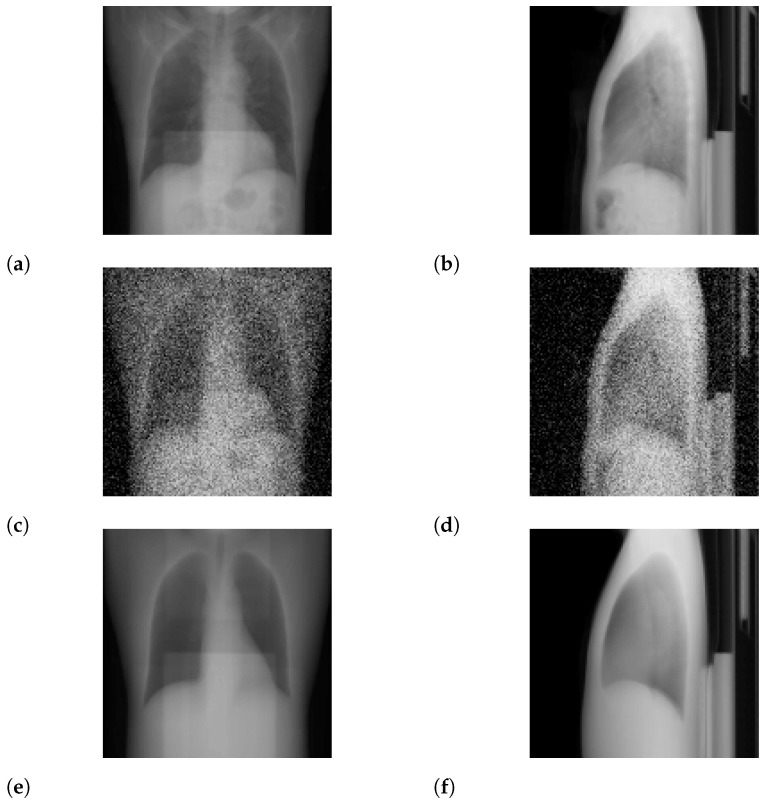
Input images and projections of a reconstructed image (N=2): (**a**,**b**) projections of the ground-truth image, (**c**,**d**) noisy input 2D images assuming an ultra-low-dose protocol, (**e**,**f**) projections of the optimum reconstructed image. The intensities of these images were modified to enhance visibility.

**Figure 8:**
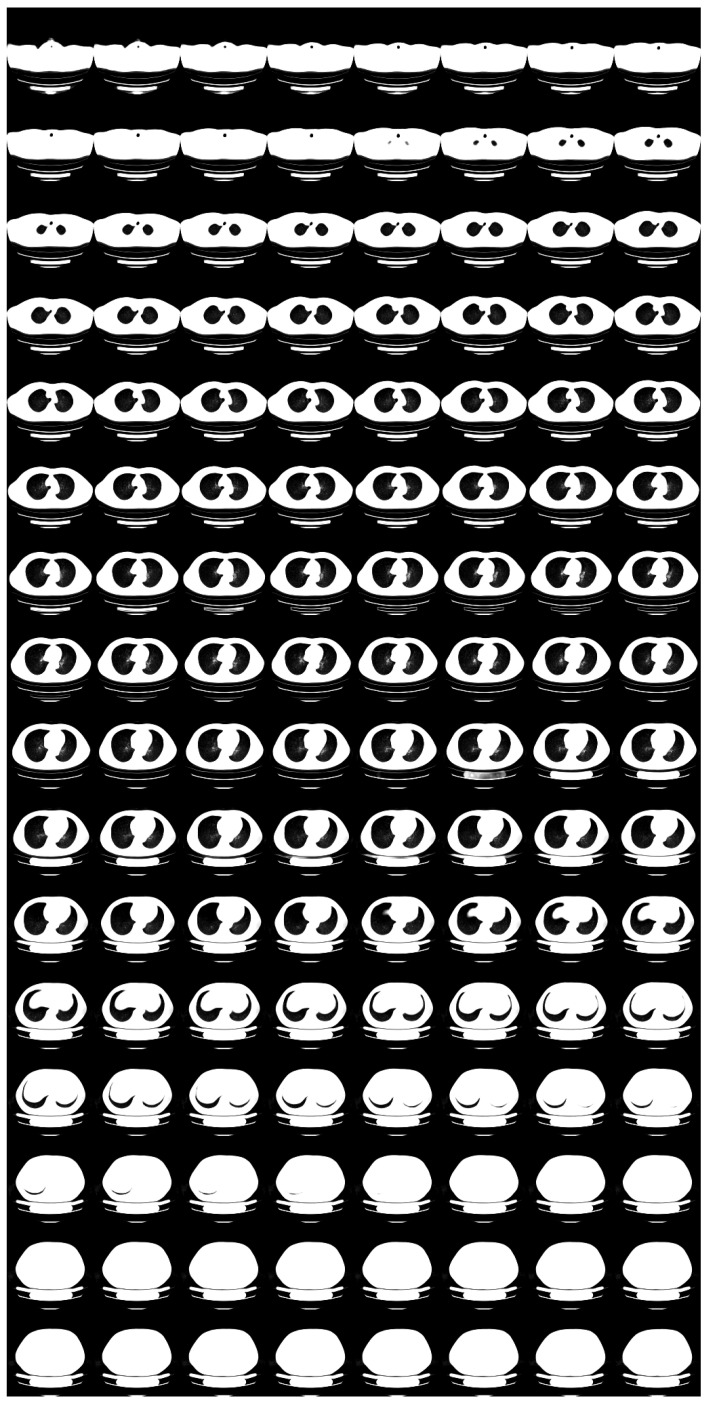
Reconstructed 3D CT image with X2CT-FLOW from Figure 7c,d ($\sigma^{2}=100,N=2$), in a pulmonary window setting.

**Figure 9:**
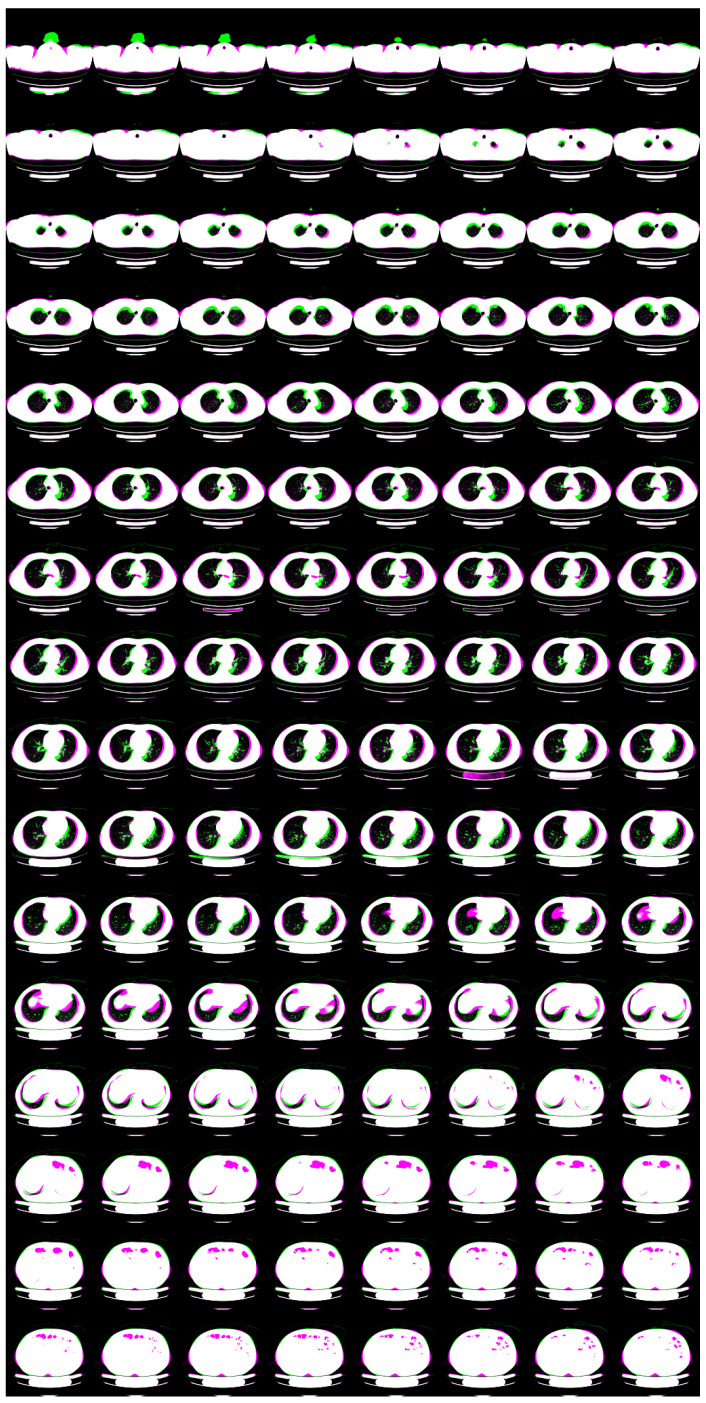
Superposition of the reconstructed 3D CT image shown in Figure 8 (magenta) and the ground-truth image (green).

For low-dose data, a noise which follows the Laplacian distribution and the normal distribution is superimposed on those data [16]. To simulate an ultra-low-dose protocol, we only added an independent normal noise $N(0,10^{2})$ to each 2D projection image $y_{i}^{j}$. We optimized Equation (10) with $\sigma^{2}=100$ and $\alpha =0.9\cdot \left[1-\exp\left(-0.01\cdot n\right)\right]$. We iterated while n≤1000 and $∥y-PG_{\theta }\left(z\right){∥}_{2}^{2}>3^{2}\cdot N\cdot H_{2D}\cdot W_{2D}$. For N=2, we show noisy input 2D images and 2D projection images of a 3D reconstructed image in Figure 7. Moreover, we show a 3D chest CT image reconstructed from Figure 7c,d in Figure 8, and a differential image between the reconstructed 3D image and the ground-truth image in Figure 9. We show enlarged axial and coronal slices in a pulmonary window setting in Figure 6. For N=1 and N=2, we show the means and variances of SSIM, PSNR, MAE, and NRMSE between the reconstructed 3D chest CT images and ground-truth images in Table 4 and Table 5. Moreover, we show our results with X2CT-GAN [6] trained with our materials explained in Section 2.1.

## 4. Discussion

We designed X2CT-FLOW to find the optimum 3D chest CT image with MAP reconstruction. We realized X2CT-FLOW by exploiting two features of the flow-based deep generative models: they can estimate the exact log-likelihood of an image, i.e., density estimation, and they can efficiently sample fictional but realistic images, i.e., sampling. Unlike in related works for 2D images [17,18,19,20,21], we reconstructed 3D CT images from 2D projection images.

We can compare the reconstruction performance (SSIM, PSNR, etc.) of X2CT-FLOW with that of X2CT-GAN [6] using the same dataset. From Table 2, Table 3, Table 4 and Table 5, we observed that those metrics are comparable. However, we stress that we achieved this performance in an unsupervised manner without especially customized deep neural networks for supervised learning.

In the limit of $\sigma^{2}\to 0$, X2CT-FLOW finds 3D chest CT images whose projections onto each 2D plane are equivalent to each original input 2D projection image with the latent space exploration (Equation (13)). The flow-based deep generative models tend to map a random vector in the latent space into a meaningful image in the distribution for training images. Although this does not guarantee that the obtained solution is in the distribution, we empirically found that our method leads to statistically meaningful solutions. Previous studies [4,5,6,7] contain the cycle consistency loss for end-to-end supervised deep learning, but those losses are for training, hence, not for testing. From this viewpoint, a related work is PULSE [22], but it deals with super-resolution between 2D images. X2CT-FLOW deals with the reconstruction of optimum 3D chest CT images from a single or a few 2D projection images.

In the standard-dose protocol, while the initial guess images (Figure 3c,d) are clearly different from the input images (Figure 3a,b), the optimum reconstructed images (Figure 3e,f) well coincide with the input images. Figure 4 and Figure 6 show that X2CT-FLOW can reconstruct the structure of organs (e.g., lungs, heart, and liver). Moreover, X2CT-FLOW can well reconstruct the position of the bed. However, X2CT-FLOW cannot well reconstruct finer structures, e.g., bronchovascular. This implies that abnormalities such as bronchovascular ones are not visible in the present reconstruction method. This issue also could impact SSIM, PSNR, MAE, and NRMSE.

We only compared our results with X2CT-GAN [6]. Comparison with other models, such as conventional and supervised learning methods, will be included in our future works, but we expect that conventional CT reconstruction algorithms could require hundreds of X-ray projection images to obtain meaningful results. It should also be noted that we did not adopt authentic CT images taken with ultra-low-dose protocols in this study.

There are five possible extensions for X2CT-FLOW. First, we emulated CT images in an ultra-low-dose protocol using normal noise, but it is required to use authentic CT images in an ultra-low-dose protocol to adopt X2CT-FLOW in clinical practice. Second, we adopted the linear operator to take an average to obtain 2D projection images from a 3D chest CT image. We can replace the linear operator with an arbitrary nonlinear differentiable operator from a 3D image to other images. Moreover, we do not have to retrain the flow-based deep generative model when we change the operator. Third, we limited the maximum number of projections for a 3D CT image to two planes (N=2), i.e., projections onto the sagittal and coronal planes. However, it is possible to increase the number of projections if additional projection images are available. This could contribute to enhancing SSIM, PSNR, MAE, and NRMSE, but it also enhances the radiation exposure. Fourth, apart from 3D GLOW, our proposed method could be applied to other kinds of flow-based deep generative model, e.g., Flow++ [23] and residual flows [24] if we extend those 2D models to 3D models. Lastly, although we adopted the dataset of normal subjects, models trained with a dataset of abnormal subjects could be used to reconstruct 3D chest CT images with abnormalities.

Although we dealt with the reconstruction of 3D chest CT images from clean or noisy 2D projection images, we can adopt the proposed algorithm to other applications apart from medical image analysis. For example, we could apply X2CT-FLOW to estimate 3D shock wave structures from 2D Schlieren images, which are projection images of the air density gradient.

## 5. Conclusions

We proposed X2CT-FLOW built upon 3D GLOW for the MAP reconstruction of 3D chest CT images from a single or a few projection images. To realize the practical high-resolution model, we recently developed progressive learning. We validated X2CT-FLOW by two numerical experiments assuming a standard-dose protocol or an ultra-low-dose protocol. The 3D chest CT images reconstructed from biplanar projection images without noise contamination showed good agreement with ground-truth images in terms of SSIM (0.7675 on average), PSNR (25.89 dB on average), MAE (0.02364 on average), and NRMSE (0.05731 on average). Moreover, our images reconstructed from images contaminated with normal noise ($N(0,10^{2})$) and the ground-truth images also showed good agreement in terms of SSIM (0.7008 on average), PSNR (23.58 dB on average), MAE (0.02991 on average), and NRMSE (0.07349 on average). Further validations of X2CT-FLOW to adopt it for clinical practice are necessary, e.g., (i) validation for the reconstruction of abnormal lesions and (ii) validation using authentic CT images in an ultra-low-dose protocol, which are included in our future works.

## Figures and Tables

**Table 1:** Hyperparameters used to train 3D GLOW model.

Flow coupling	Affine
Learn-top option	True
Flow permutation	1 × 1 × 1 convolution
Minibatch size	1 per GPU
Train epochs	96 (2 bits)
	324 (3 bits from 2 bits)
	24 (4 bits from 3 bits)
	144 (8 bits from 4 bits)
Layer levels	5
Depth per level	8
Filter width	512
Learning rate in steady state	$1.0\times 10^{-4}$

**Table 2:** Means of metrics (N=1, standard-dose protocol). We show variances in brackets.

Method	Ours	X2CT-GAN [6]
SSIM	0.4897 (0.00437)	0.5349 (0.001257)
PSNR [dB]	17.57 (4.755)	19.53 (1.152)
MAE	0.08299 (0.001008)	0.005758 (6.17 × 10${}^{-7}$)
NRMSE	0.1374 (0.002066)	0.1064 (0.0001714)

**Table 3:** Means of metrics (N=2, standard-dose protocol). We show variances in brackets.

Method	Ours	X2CT-GAN [6]
SSIM	0.7675 (0.001931)	0.7543 (0.0005110 )
PSNR [dB]	25.89 (2.647)	25.22 (0.5241)
MAE	0.02364 ($5.645\times 10^{-5}$)	0.02648 (5.552 × 10^−6^)
NRMSE	0.05731 (0.0002204)	0.05502 (2.181 × 10^−5^)

**Table 4:** Means of metrics (N=1, ultra-low-dose protocol). We show variances in brackets.

Method	Ours	X2CT-GAN [6]
SSIM	0.4989 (0.000536)	0.5151 (0.001028)
PSNR (dB)	18.16 (0.1560)	19.38 (0.9493)
MAE	0.07480 (2.98 × 10^−5^)	0.005943 ($5.53\times 10^{-7}$)
NRMSE	0.1237 (3.20 × 10^−5^)	0.1081 (0.0001485)

**Table 5:** Means of metrics (N=2, ultra-low-dose protocol). We show variances in brackets.

Method	Ours	X2CT-GAN [6]
SSIM	0.7008 (0.0005670)	0.6828 (0.0002700)
PSNR (dB)	23.58 (0.6132)	23.78 (0.2827)
MAE	0.02991 ($1.052\times 10^{-5}$)	0.03251 (4.193 × 10^−6^)
NRMSE	0.07349 ($5.007\times 10^{-5}$)	0.06486 (1.607 × 10^−5^)

**Figure A1:**
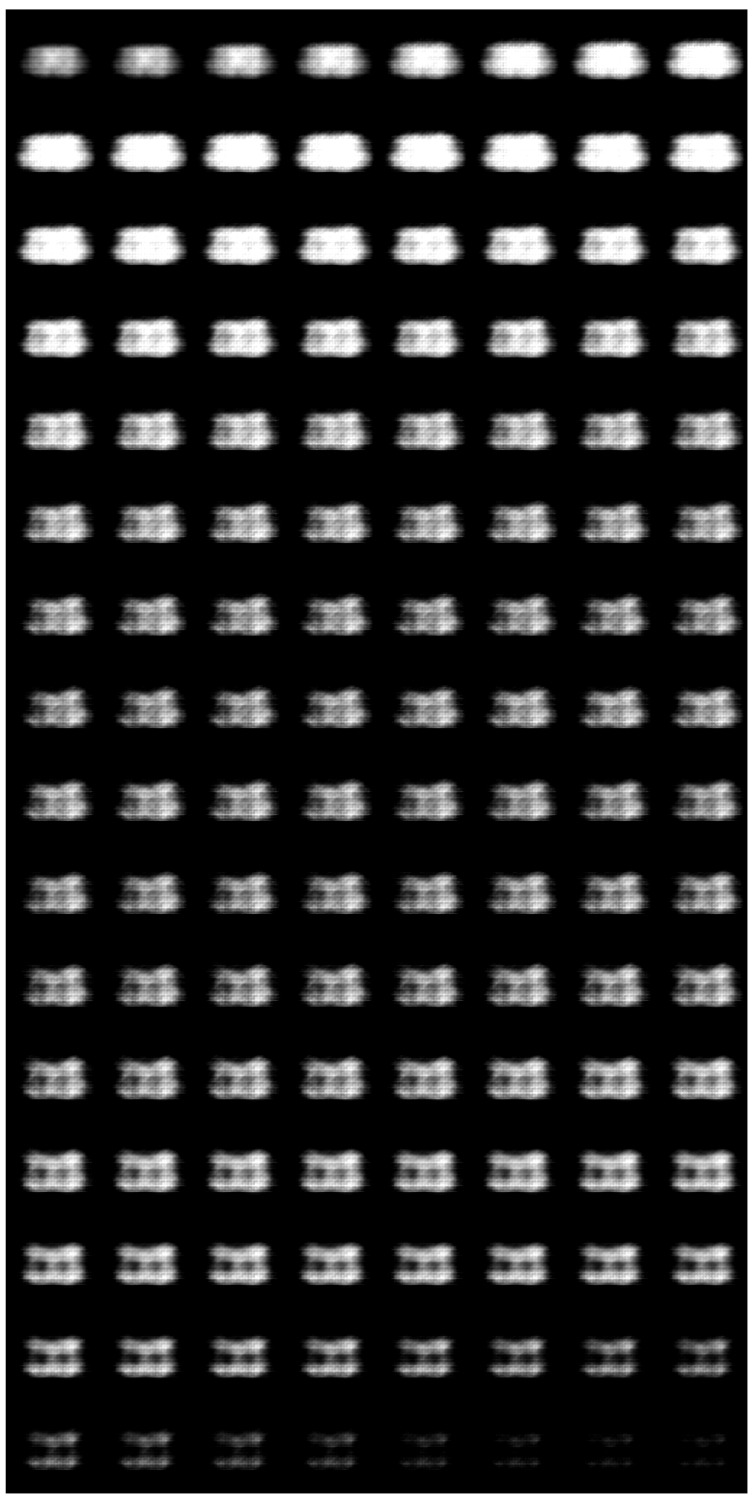
Fictional mean 3D CT image at 48 epochs (sampled with T=0, standard learning), in pulmonary window setting.

**Figure A2:**
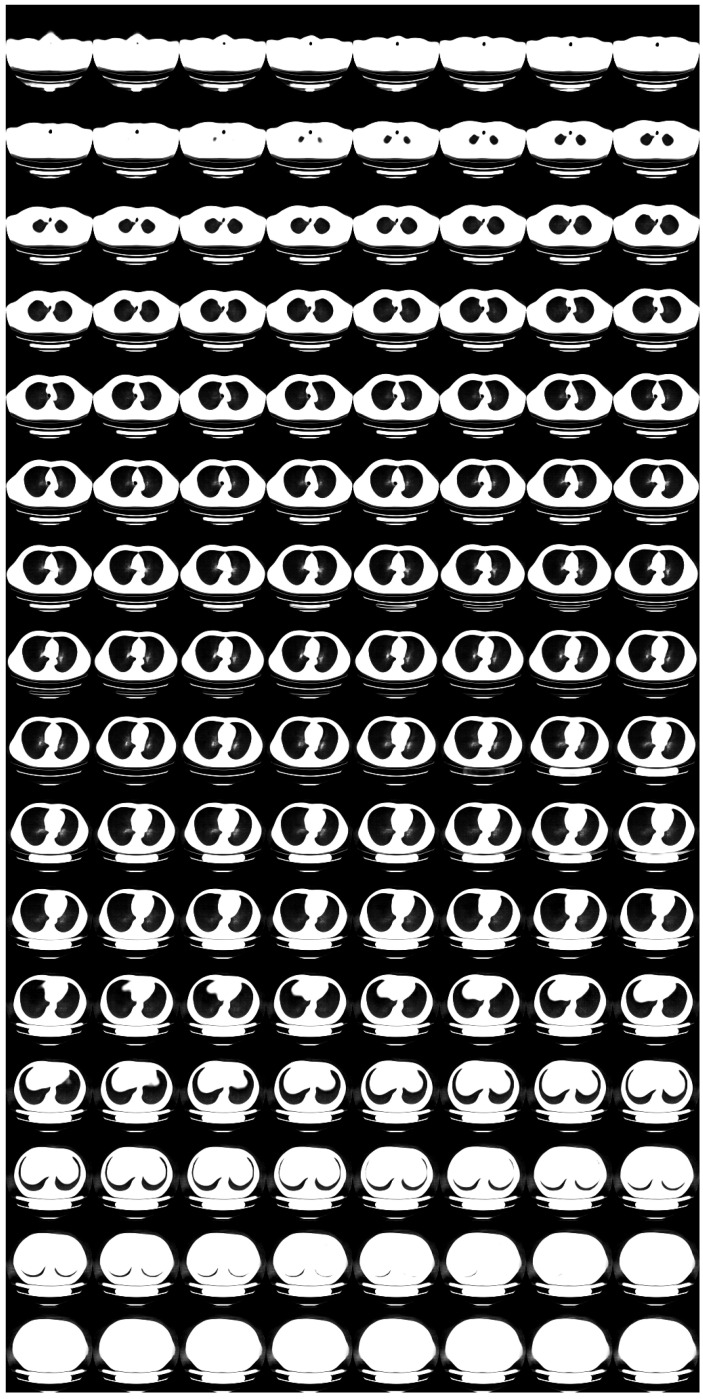
Fictional mean 3D CT image at the final epochs (sampled with T=0, progressive learning), in pulmonary window setting.

**Figure A3:**
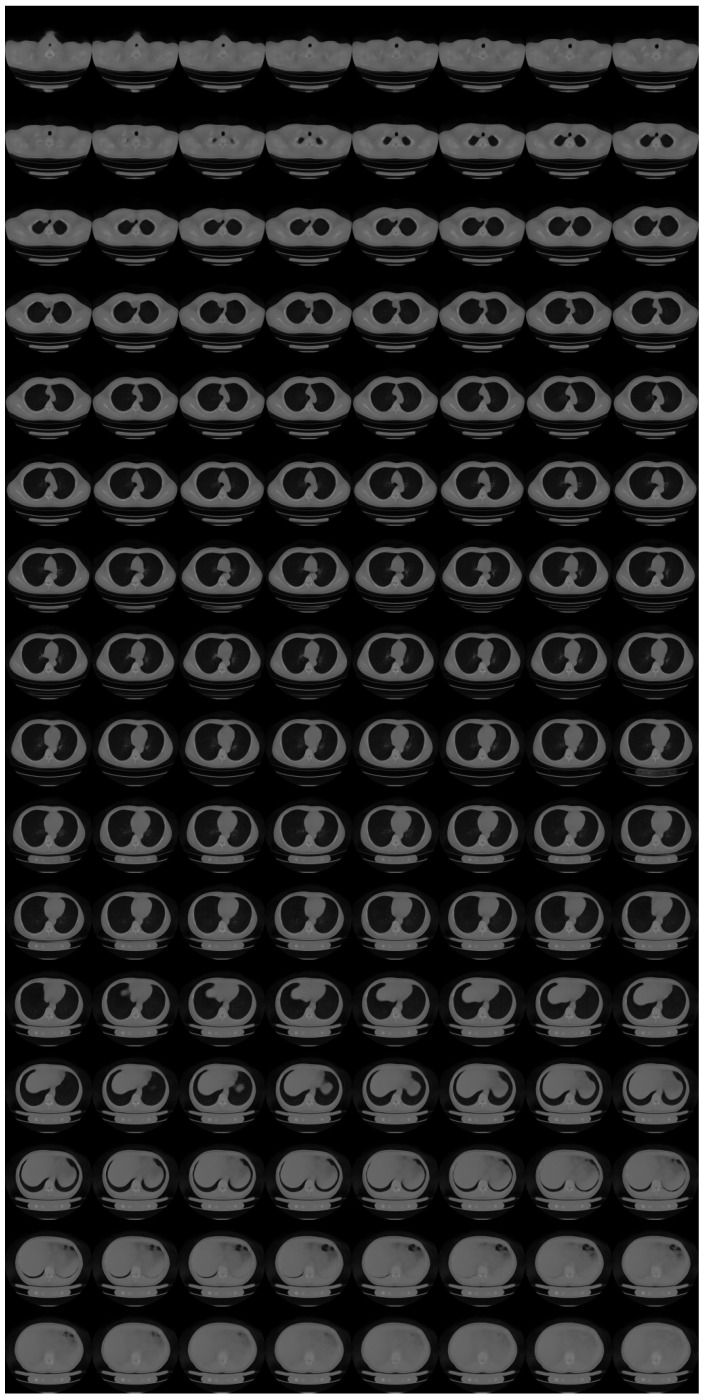
Fictional 3D CT image at the final epochs (sampled with T=0.5, progressive learning, 1 of 3).

**Figure A4:**
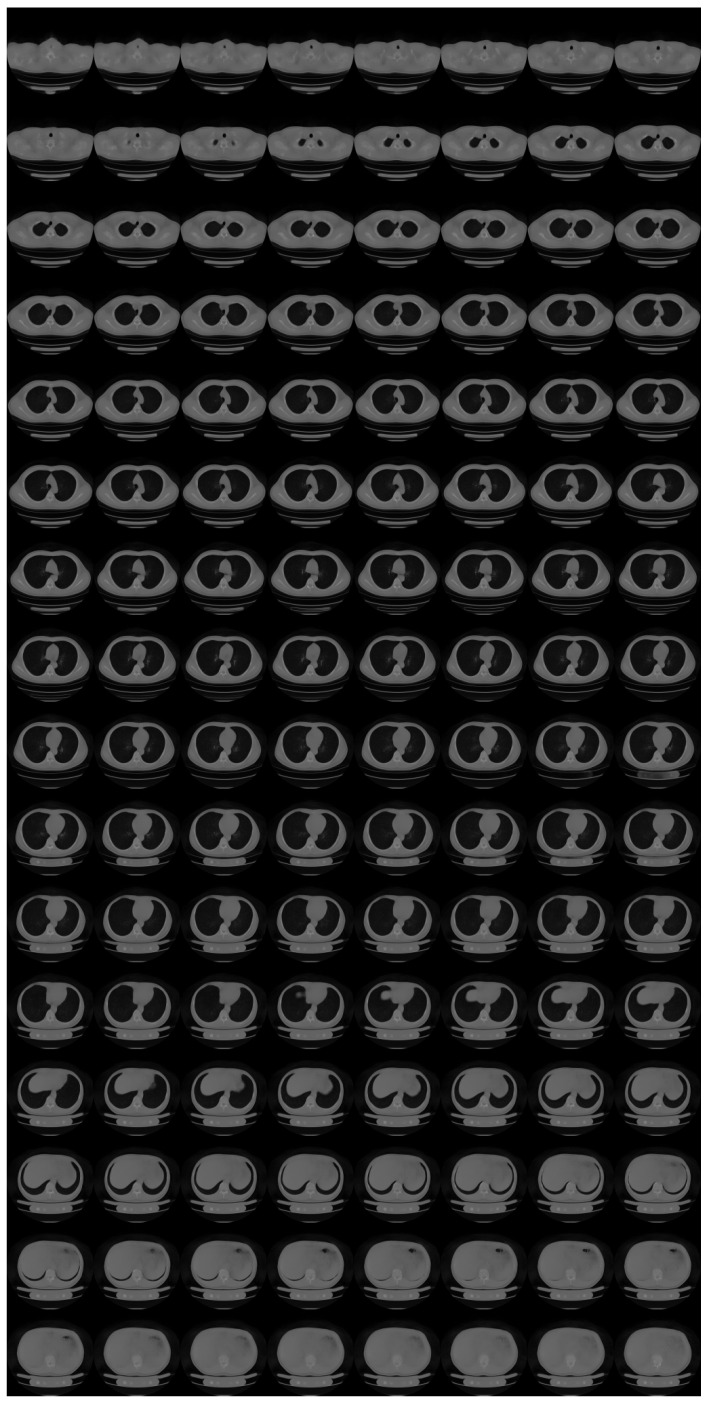
Fictional 3D CT image at the final epochs (sampled with T=0.5, progressive learning, 2 of 3).

**Figure A5:**
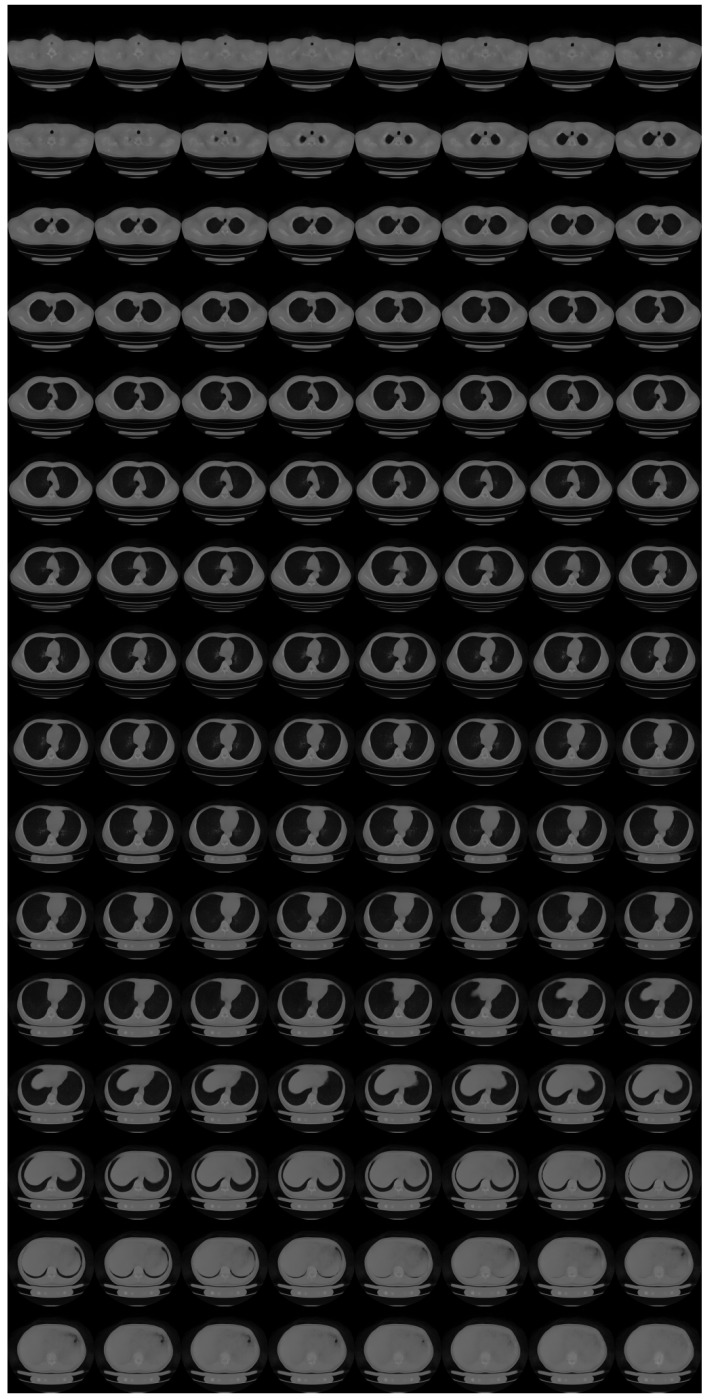
Fictional 3D CT image at the final epochs (sampled with T=0.5, progressive learning, 3 of 3).

## Data Availability

The dataset of computed tomography images is protected under the laws of our institution; hence, it is not open to the public.

## Abbreviations

    The following abbreviations are used in this manuscript:

CT	computed tomography
MAP	maximum a posteriori
2D	two-dimensional
3D	three-dimensional
PSNR	peak signal-to-noise ratio
SSIM	structural similarity
MAE	mean absolute error
NRMSE	normalized root mean square error
CXR	chest X-ray

## Appendix A. Details for Progressive Learning

In our progressive learning, we begin to train the whole dataset at lower color gradations, e.g., 2 bits. If the validation loss takes its local minima, we restart to train the whole dataset at higher color gradations, e.g., 8 bits. The required time to obtain the same negative log-likelihood decreases if we adopt the progressive learning. Note that our progressive learning reduces image color gradations in the training dataset as a pre-processing before the dequantization as in, e.g., NICE [13].

Finally, we show our code for reducing image color gradations.

**import** numpy as np
# *src : source image (ndarray)*
# *n_bits_dst : the number of bits for the destination image*
# *(integer)*
# *n_bits_src : the number of bits for the source image*
# *(integer)*
# *return : destination image with reduction (ndarray)*
**def** color_reduction(src, n_bits_dst = 4, n_bits_src = 8):
       dst = np.copy(src)
       delta = 2∗∗n_bits_src // 2∗∗n_bits_dst
       **for** c **in** **range**(2∗∗n_bits_src // delta):
          inds = np.where((delta ∗ c <= src) \
                                     & (delta ∗ (c + 1) > src))
          dst[inds] = (2 ∗ delta ∗ c + delta ) // 2
       **return** dst

## Appendix B. Ablation Study for Progressive Learning

We abruptly started training the 3D chest CT model with 8 bits and continued it until 588 (=96 + 324 + 24 + 144) epochs. We validated the model once per 12 epochs. The validation loss (NLL: negative log-likelihood) took its minimum value at 48 epochs. Figure A1 and Figure A2 show sampling results with T=0 for this standard learning at 48 epochs where we experienced a local minima for NLL (bit per dimension =2.188) and the progressive learning at the final epochs and when the model experienced the minimum NLL (bit per dimension =1.827) in the 8 bits training, respectively. Furthermore, we show sampling results with T=0.5 for the progressive learning in Figure A3, Figure A4 and Figure A5. These figures apparently show the superiority of the progressive learning. Specifically, the images generated using the progressive learning contain more anatomical features than those generated using the standard learning.

## Appendix C. Formulations for *N* ≥ 2

We define other projection operators $P^{j}$, variances ${\left(\sigma^{j}\right)}^{2}$, projection images $y^{j}$, and noise vector $w^{j}$. We distinguish projection directions by the superscript *j*. We assume that there is no correlation among $w^{j}$. Therefore, we have

(A1) $$\begin{array}{ccc} y^{j}-P^{j}x & = & \sqrt{{\left(\sigma^{j}\right)}^{2}}w^{j}, \end{array}$$

(A2) $$\begin{array}{ccc} w^{j} & ∼ & N(0,I). \end{array}$$

The log-posterior is now conditioned with all those projection images $y^{j}$. Therefore, we have

$$\begin{array}{ccc} \hat{x} & = & \underset{x}{\arg\;\max}\log p\left(x|y^{1},y^{2},\ldots ,y^{N}\right)\\ & = & \underset{x}{\arg\;\max}\log p\left(y^{1},y^{2},\ldots ,y^{N}|x\right)+\log p\left(x\right)\\ & = & \underset{x}{\arg\;\max}\sum_{j}\log p\left(y^{j}|x\right)+\log p\left(x\right)\\ & = & \underset{x}{\arg\;\max}\sum_{j}\log\left[\frac{1}{\sqrt{2\pi {\left(\sigma^{j}\right)}^{2}}}\exp\left(-\frac{1}{2}{\left(w^{j}\right)}^{T}w^{j}\right)\right]+\log p\left(x\right) \end{array}$$

(A3) $$\begin{array}{ccc} & = & \underset{x}{\arg\;\max}\sum_{j}-\frac{1}{2{\left(\sigma^{j}\right)}^{2}}{∥y^{j}-P^{j}x∥}_{2}^{2}+\log p\left(x\right) \end{array}$$

(A4) $$\begin{array}{ccc} & \equiv & \underset{x}{\arg\;\max}-E^{\prime }\left(x\right). \end{array}$$

In the deformation from the second line to the third line, we applied the fact that normal noise distributions among $y^{j}$ are independent of each other.

## References

[1] Nam J.G., Ahn C., Choi H., Hong W., Park J., Kim J.H., Goo J.M. (2021). Image quality of ultralow-dose chest CT using deep learning techniques: Potential superiority of vendor-agnostic post-processing over vendor-specific techniques. Eur. Radiol..

[2] Levitan E., Herman G.T. (1987). A maximum a posteriori probability expectation maximization algorithm for image reconstruction in emission tomography. IEEE Trans. Med. Imaging.

[3] Baraniuk R.G. (2007). Compressive sensing [lecture notes]. IEEE Signal Process. Mag..

[4] Shen L., Zhao W., Capaldi D., Pauly J., Xing L. (2021). A Geometry-Informed Deep Learning Framework for Ultra-Sparse 3D Tomographic Image Reconstruction. arXiv.

[5] Shen L., Zhao W., Xing L. (2019). Patient-specific reconstruction of volumetric computed tomography images from a single projection view via deep learning. Nat. Biomed. Eng..

[6] Ying X., Guo H., Ma K., Wu J., Weng Z., Zheng Y. X2CT-GAN: Reconstructing CT from biplanar X-rays with generative adversarial networks. Proceedings of the IEEE/CVF Conference on Computer Vision and Pattern Recognition.

[7] Peng C., Liao H., Wong G., Luo J., Zhou S.K., Chellappa R. (2020). XraySyn: Realistic View Synthesis From a Single Radiograph Through CT Priors. arXiv.

[8] Henzler P., Rasche V., Ropinski T., Ritschel T. (2018). Single-image Tomography: 3D Volumes from 2D Cranial X-Rays. Computer Graphics Forum.

[9] Shibata H., Hanaoka S., Nomura Y., Nakao T., Sato I., Sato D., Hayashi N., Abe O. (2021). Department of Radiology, The University of Tokyo Hospital, 7-3-1 Hongo, Bunkyo-ku, Tokyo, 113-8655, Japan. Search articles by ’Osamu Abe’ Abe O. Versatile anomaly detection method for medical images with semi-supervised flow-based generative models. Int. J. Comput. Assist. Radiol. Surg..

[10] Kobyzev I., Prince S., Brubaker M. (2020). Normalizing flows: An introduction and review of current methods. IEEE Trans. Pattern Anal. Mach. Intell..

[11] Kingma D.P., Dhariwal P. (2018). Glow: Generative flow with invertible 1x1 convolutions. arXiv.

[12] Parmar N., Vaswani A., Uszkoreit J., Kaiser L., Shazeer N., Ku A., Tran D. Image transformer. Proceedings of the International Conference on Machine Learning.

[13] Dinh L., Krueger D., Bengio Y. (2014). Nice: Non-linear independent components estimation. arXiv.

[14] Dinh L., Sohl-Dickstein J., Bengio S. (2016). Density estimation using real nvp. arXiv.

[15] Wang Z., Bovik A.C., Sheikh H.R., Simoncelli E.P. (2004). Image quality assessment: From error visibility to structural similarity. IEEE Trans. Image Process..

[16] Zeng D., Huang J., Bian Z., Niu S., Zhang H., Feng Q., Liang Z., Ma J. (2015). A simple low-dose X-ray CT simulation from high-dose scan. IEEE Trans. Nucl. Sci..

[17] Kothari K., Khorashadizadeh A., de Hoop M., Dokmanić I. (2021). Trumpets: Injective Flows for Inference and Inverse Problems. arXiv.

[18] Asim M., Daniels M., Leong O., Ahmed A., Hand P. Invertible generative models for inverse problems: Mitigating representation error and dataset bias. Proceedings of the International Conference on Machine Learning.

[19] Whang J., Lei Q., Dimakis A.G. (2020). Compressed sensing with invertible generative models and dependent noise. arXiv.

[20] Whang J., Lindgren E., Dimakis A. Composing Normalizing Flows for Inverse Problems. Proceedings of the International Conference on Machine Learning.

[21] Marinescu R.V., Moyer D., Golland P. (2020). Bayesian Image Reconstruction using Deep Generative Models. arXiv.

[22] Menon S., Damian A., Hu S., Ravi N., Rudin C. PULSE: Self-supervised photo upsampling via latent space exploration of generative models. Proceedings of the IEEE/CVF Conference on Computer Vision and Pattern Recognition.

[23] Ho J., Chen X., Srinivas A., Duan Y., Abbeel P. Flow++: Improving flow-based generative models with variational dequantization and architecture design. Proceedings of the International Conference on Machine Learning.

[24] Chen R.T., Behrmann J., Duvenaud D., Jacobsen J.H. (2019). Residual flows for invertible generative modeling. arXiv.

